# LIC12254 Is a Leptospiral Protein That Interacts with Integrins via the RGD Motif

**DOI:** 10.3390/tropicalmed8050249

**Published:** 2023-04-26

**Authors:** Maria F. Cavenague, Aline F. Teixeira, Luis G. V. Fernandes, Ana L. T. O. Nascimento

**Affiliations:** 1 Laboratório de Desenvolvimento de Vacinas, Instituto Butantan, São Paulo 05503-000, SP, Brazil; 2 Programa de Pós-Graduação Interunidades em Biotecnologia, Instituto de Ciências Biomédicas, Universidade de São Paulo, São Paulo 05508-900, SP, Brazil

**Keywords:** *Leptospira*, leptospirosis, integrins, RGD

## Abstract

Pathogenic leptospires can bind to receptors on mammalian cells such as cadherins and integrins. *Leptospira* effectively adheres to cells, overcomes host barriers and spreads into the bloodstream, reaching internal target organs such as the lungs, liver and kidneys. Several microorganisms produce proteins that act as ligands of integrins through the RGD motif. Here, we characterized a leptospiral RGD-containing protein encoded by the gene *lic12254.* In silico analysis of pathogenic, intermediate and saprophytic species showed that LIC12254 is highly conserved among pathogenic species, and is unique in presenting the RGD motif. The LIC12254-coding sequence is greatly expressed in the virulent *Leptospira interrogans* L1-130 strain compared with the culture-attenuated *L. interrogans* M20 strain. We also showed that the recombinant protein rLIC12254 binds to αVβ8 and α8 human integrins most likely via the RGD motif. These interactions are dose-dependent and saturable, a typical property of receptor–ligand interactions. The binding of the recombinant protein lacking this motif—rLIC12254 ΔRAA—to αVβ8 was almost totally abolished, while that with the α8 human integrin was decreased by 65%. Taken together, these results suggest that this putative outer membrane protein interacts with integrins via the RGD domain and may play a key role in leptospirosis pathogenesis.

## 1. Introduction

Leptospirosis is a worldwide zoonosis caused by pathogenic spirochetes of the genus *Leptospira*. Globally disseminated, over 1 million infections occur annually around the world, with higher prevalence in tropical and subtropical countries [1]. The infection in humans occurs through the host’s mucosa or broken skin via direct contact with the urine of infected animals or by indirect contact with contaminated water or soil. The infectious process causes a wide range of clinical symptoms. The initial phase is characterized by mild flu-like symptoms such as fever, chills, headache and nausea, which may lead to a misleading diagnosis of other febrile illnesses. However, the evolution of the disease may cause severe conditions, resulting in Weil’s syndrome, pulmonary hemorrhage and renal and hepatic failure, with a mortality rate of up to 50% [2].

Understanding the pathogenic mechanisms of *Leptospira* is extremely important for the effective development of vaccines and diagnostic tests. Outer membrane proteins are considered potential antigens since they play a vital role in mediating interactions with extracellular matrix components, serum components, mammalian cells and cell receptors such as cadherins and integrins. Thus, the characterization of outer membrane proteins is an interesting strategy for elucidating the pathogenetic mechanism of *Leptospira* spp. [3].

It is well known that pathogenic leptospires are effective in adhering and crossing the host barriers to then spread through blood vessels, aiming to reach target organs such as the lungs, liver and kidney [2]. Possibly, surface-exposed proteins are responsible for mediating this interaction. In the last years, several leptospiral proteins including LipL32, Lsa24, OmpL37, OmpL1, LipL41, LipL21, LigA and LigB have been identified as adhesins with the ability to bind to fibronectin, laminin, vascular elastin and collagens [4,5,6,7,8,9]. More recently, LipL41 and OmpL1 were characterized as being able to interact with a variety of endothelial, epithelial and fibroblast cell lines, while OmpL37, LipL21 and LipL46 interact exclusively with HEK293T renal proximal tubule epithelial cells [10].

The ability of pathogenic leptospires to bind to receptors such as cadherins and integrins of mammalian cells has already been investigated [11]. In the case of CR3 integrin, its recognition does not seem to occur via the RGD motif, but a fibronectin molecule adsorbed on the *Leptospira* surface seems to be necessary for that interaction [12]. The ligand responsible for mediating this interaction has not yet been characterized. In 2012, Zhang and collaborators identified the leptospiral Mce protein as a virulent factor able to interact with the β1, β2 and β3 integrin families via the RGD (Arg–Gly–Asp) motif present in this protein [13]. *L. interrogans* serovar Copenhageni genome sequences have approximately 102 coding sequences (CDS) for proteins containing one or more RGD motifs. Among these sequences, around 24 are described as putative proteins, hypothetical proteins, and outer membrane proteins. Previously performed in silico analysis showed LIC12254 as an outer membrane protein. Thus, as the interaction between infectious ligands containing the RGD motif and integrins in the host plays an important role in the invasion of pathogens, the aim of present study was to investigate whether LIC12254 leptospiral protein is able to bind to human integrins. Here, we show that the outer membrane protein encoded by the gene *lic12254* is expressed in high amounts in low-passage pathogenic leptospires, contains an RGD motif, which present high similarity with other pathogenic species and binds to the αVβ8 and α8 human integrins via the RGD motif.

## 2. Materials and Methods

### 2.1. Bioinformatics Analysis of LIC12254

The LIC12254 coding sequence was analyzed using the LipoP for prediction of SPI (Signal Peptidase I) cleavage site [14] and PRED-TMBB [15] and Interprot [16] web servers for prediction of β-barrel topology and conserved domain. Finally, the protein localization was predicted by the CELLO program [17].

### 2.2. Protein Sequence Alignment

The amino-acid sequences of potential protein orthologs from 20 *Leptospira* species, representing pathogenic, intermediate and saprophytic strains, were recovered from the NCBI database using the Basic Local Alignment Search Tool (BLAST) and protein–protein BLAST (BLASTp) analysis against the non-redundant database [18]. The following species were analyzed: the pathogenic species (P1) *L. interrogans* serovar Copenhageni L1-130, *L. kirschneri*, *L. noguchii, L. alstonii, L. weilii, L. santarosai*, *L. alexanderi, L. kmetyi*, *L. borgpetersenii*; the intermediate species (P2) *L. licerasiae*, *L. fainei, L. broomii, L. inadai, L. wolffii*; and the saprophytic species (S1) *L. biflexa*, *L. meyeri*, *L. terpstrae*, *L. wolbachii*, *L. vanthielii* and *L. yanagawae*. The phylogenetic relationships were built with sequences obtained in this study, using the Neighbor-Joining method and Poison model, using Mega11 software [19]. Node support for the resulting phylogenetic tree was assessed by 1000 bootstrap replicates to define the robustness of the findingy [20]. Aiming to search for the conserved amino acids in the RGD motif present in the LIC12254, an alignment was performed using the PROMALS3D alignment program [21]. Table 1 contains all the Species taxIDs and Sequence IDs used in this study.

### 2.3. Predicted Three-Dimensional (3D) Structure

To investigate the position of the RGD motif in the structure of the protein coded by LIC12254, the 3D structure was modeled by submitting the linear sequences to the I-TASSER [22], C-I-TASSER [23], TrRosetta [24] web servers and the AlphaFold2 protein structures database [25]. The models with the best confidence score and Z-score were chosen and then visualized using the PyMOL molecular graphics system for all sequences (http://www.pymol.org/pymol (accessed on 22 July 2022)). Highlighting the RGD motif, also using PyMOL software, showed the predicted positions of the three amino acids.

### 2.4. Bacterial Strain

The pathogenic bacteria *L. interrogans* serovar Copenhageni strain Fiocruz L1-130 and the culture-attenuated *L. interrogans* serovar Copenhageni strain M20 were grown in liquid EMJH medium (Difco) supplemented with 10% Leptospira Enrichment EMJH (Difco) under aerobic conditions. All cultures were maintained in the Faculdade de Medicina Veterinária e Zootecnia, USP, São Paulo, SP, Brazil. The strains of *E. coli* DH5a and *E. coli* BL21 (DE3) Star pLysS (Invitrogen, Waltham, MA, USA) were used for cloning and protein expression, respectively.

### 2.5. RNA Extraction and Real-Time Reverse-Transcription PCR (RT-qPCR)

Leptospiral cells were recovered from the liquid EMJH culture medium by centrifugation (3075× *g*, 15 min, 4 °C), and total RNA was extracted using the Trizol reagent (Invitrogen), as recommended by the manufacturer. To eliminate the residual DNA, the samples were incubated with DNAse I (Invitrogen). A reverse-transcription PCR amplification of RNAs was performed to obtain cDNAs by using the SuperScript III kit Reverse Transcriptase (Invitrogen). RT-qPCR was performed using CFX96 RealTime System (Bio-Rad, Hercules, CA, USA) and SYBR Green I dye (Applied Biosystems, Waltham, MA, USA) to detect the synthesized double-stranded DNAs, using the primer pairs described in Table 2. The reactions were carried out with SYBR Green PCR Master Mix (Applied Biosystems) in a 20 μL reaction volume, using the following cycle parameters: 95 °C for 10 min, 40 cycles of 95 °C for 15 s and 58 °C for 1 min. The relative gene expression among leptospiral strains was calculated using comparative 2^−ΔΔCt^ [26].

### 2.6. Amplification of the Gene lic12254

The gene *lic12254* was amplified without the signal peptide (from aa 35) from the genomic DNA of *L. interrogans* by PCR with specific primers (Table 2). The PCR products were purified using the GFX™ PCR DNA and Gel Band Purification kit (GE Healthcare, Chicago, IL, USA) and cloned into the expression vector pAE at the restriction sites (*XhoI* and *KpnI)*. This vector allows the expression of recombinant proteins containing a sequence of 6 histidine residues in the N-terminal region [27] and has a high expression system controlled by the T7 phage promoter. The constructs were analyzed by DNA sequencing using the T7 primers with an ABI sequencer (PE Applied Biosystems).

### 2.7. Construction of a lic12254 Gene Having a Mutated RGD Motif

The RGD motif located at the amino acid position 153–155 in the LIC12254 protein sequence of wild-type *L. interrogans* serovar Copenhageni was mutated by PCR. The RGD motif (Arg–Gly–Asp) was replaced by RAA (Arg–Ala–Ala). Two codons (GGG and GAT), encoding glycine and aspartic acid (GD), were replaced with two other codons (GCT and GCG), encoding two alanine residues (AA). The gene was divided into two parts, the first (R1) from the beginning of the gene to the region to be replaced, and the second (R2) from the region to be replaced to the end of the gene. First, R1 was amplified using a forward oligonucleotide (LIC12254 F) and a reverse oligonucleotide (ΔRAA R) that contained the motif sequence substitution (Table 3). R2 was amplified using a forward oligonucleotide (ΔRAA F) that contained the motif replacement and a sequence complementary to the end of the R1 PCR product and a reverse oligonucleotide (LIC12254 R). The PCR products generated from both amplifications (R1 and R2) were used as templates for another PCR round, this time using a forward oligonucleotide (LIC12254 F) complementary to the beginning of the gene and a reverse oligonucleotide (LIC12254 R) complementary to the end of the gene. Finally, the sequence of the LIC12254 protein with the RAA motif was obtained, which was named LIC12254 ΔRAA. Table 2 shows all primers used to clone the gene and the mutated one.

### 2.8. Expression of Recombinant LIC12254 and LIC12254 ΔRAA Proteins

For protein expression, pAE-LIC12254 and pAE-LIC12254 ΔRAA were introduced into *E. coli* BL21 Star (DE3) pLysS. LB medium was inoculated with 5 mL (1%) of an overnight growth culture. When an optical density (OD600_nm_) of 0.6 was achieved, the expression of the recombinant proteins was induced after the addition of 1 mM IPTG. The bacteria were collected by centrifugation, resuspended in buffer containing 10 mM Tris (pH 8.0), 150 mM NaCl, 200 μg/mL of lysozyme, 2 mM phenylmethylsulphonyl fluoride (PMSF) and 1% Triton X-100, and sonicated at 60 Hz for 10 min (2 min on, 2 min off) on ice. The soluble fraction was recovered from the supernatant of the cell lysate by centrifugation at 12,000× *g* for 10 min at 4 °C. The pellet was washed twice with buffer containing urea (10 mM Tris, pH 8.0, 150 mM NaCl, 2 M urea), and the remaining pellet was then resuspended in denaturing buffer (10 mM Tris [pH 8.0], 150 mM NaCl, 8 M urea). Finally, refolding of the denatured protein was performed by dilution with 100-fold volumes of refolding buffer (PBS [pH 7.4], 1 M urea, 10% glycerol, 0.005% Tween 20, 0.5 mM PMSF and 5 mM DTT), under agitation at 4 °C for 8 h. Both preparations (soluble and insoluble) were then dialyzed in PBS buffer containing 10% glycerol. The total volume was concentrated on Amicon Ultra Centrifugal Filters (Merck Millipore, Burlington, MA, USA) by centrifugation at 18,000× *g* at 4 °C for 20 min. The purified proteins were analyzed by SDS-PAGE and quantified by densitometry analysis using a BSA concentration curve.

### 2.9. Antiserum Production in Mice against the Recombinant Proteins

Mouse polyclonal antisera against the recombinant proteins were generated by subcutaneous immunization of female BALB/c mice (4–6 weeks old) with 10 µg of rLIC12254 adsorbed in 10% (*vol*/*vol*) Alhydrogel [2% Al(OH)_3_; Brenntag Biosector], used as an adjuvant. After two weeks, two subsequent booster injections with the same preparation were administered. Mice were injected with PBS/adjuvant, as a negative control. Two weeks after each immunization, the mice were bled from the retro-orbital plexus, and pooled sera were analyzed by enzyme-linked immunosorbent assay (ELISA, Helsinki, Finland) for the determination of antibody titers. As we used *E. coli* as the host for recombinant protein expression, the anti-recombinant-protein sera were adsorbed with a suspension of *E. coli* to suppress the possible anti-*E. coli* antibody reactivity [28]. A Western blot was performed with the anti-LIC12254 serum (1:5000) to confirm the detection of the recombinant proteins LIC12254 and LIC12254 ΔRAA.

### 2.10. Integrin Binding Test

Integrins were selected as the coating proteins in an ELISA to determine their binding to the leptospiral rLIC12254 protein, according to previous protocols [29]. Briefly, ELISA plates (Corning) were coated with 100 ng per well of α5β1, α_V_β3, α_V_β6, α_V_β8, α_V_β1, α_V_β5, αIIbβ3 or α8 integrin proteins (Santa Cruz, CA, USA) at 4 °C for 16 h. After blocking with 1% BSA–PBS and washing with PBS, 1 µg per well of rLIC12254 and rLIC12254 ΔRAA proteins was added to the plates for a 2 h incubation at 37 °C, and the plates were then washed three times with PBS containing 0.05% Tween-20 (PBS-T). Mouse anti-rLIC12254 IgG (1:2000) was used as the primary antibody, and HRP-conjugated rabbit anti-mouse IgG (1:5000) (Sigma-Aldrich, St. Louis, MO, USA) as the secondary antibody, diluted in PBS-T/BSA (1%), and the plates were incubated for 1 h at 37 °C. The wells were washed three times, and OPD (o-phenylenediamine hydrochloride) (Sigma Aldrich) (1 mg/mL) in citrate phosphate buffer (pH 5.0) plus 1 μL/mL of H_2_O_2_ was added (100 μL per well). The reaction was allowed to proceed for 15 min and then interrupted by the addition of 50 μL of 2 M H_2_SO_4_. Readings were taken at 492 nm in a microplate reader (Multiskan EX; Thermo Fisher, Waltham, MA, USA). In the test, the rOmpL1 protein derived from *L. interrogans* serovar Copenhageni provided by our laboratory [7] was used as the control.

### 2.11. Dose–Response Analysis of the Binding of the Recombinant Proteins to Human Integrin

Integrin affinity for the leptospiral rLIC12254 and rLIC12254 ΔRAA proteins was determined using ELISA as previously reported [29]. Briefly, 96-well polystyrene plates (Corning) were coated with 100 ng per well of αVβ8 or α8 integrins at 4 °C for 16 h. After blocking with 1% BSA–PBS and washing with PBS-T, increasing concentrations of the recombinant proteins were added to the plates for a 2 h incubation at 37 °C. The assessment of the bound proteins was performed by incubation for 1 h at 37 °C with the anti-rLIC12254 antibody (diluted 1:2000 in PBS-T containing 1% BSA). After washings with PBS-T, 100 μL of 1:5000-diluted HRP-conjugated rabbit anti-mouse IgG (Sigma-Aldrich) in PBS-T/BSA (1%) was added per well, followed by incubation for 1 h at 37 °C. The reaction was revealed with the OPD substrate as described above.

### 2.12. Statistical Analysis

The statistical analysis was performed by the Student’s paired t-test to determine the significance of the differences between the means. *p* < 0.05 was considered statistically significant. The results are expressed as the mean ± SD.

### 2.13. Ethics Statement

The animal studies were approved by the Ethics Committee for Animal Research of the Instituto Butantan, Brazil, under protocol no. 6226260418. The Committee for Animal Research of the Instituto Butantan adopts the guidelines of the Brazilian College of Animal Experimentation (COBEA).

## 3. Results

### 3.1. LIC12254 Characterization by Bioinformatics Analysis

**Figure 1:**
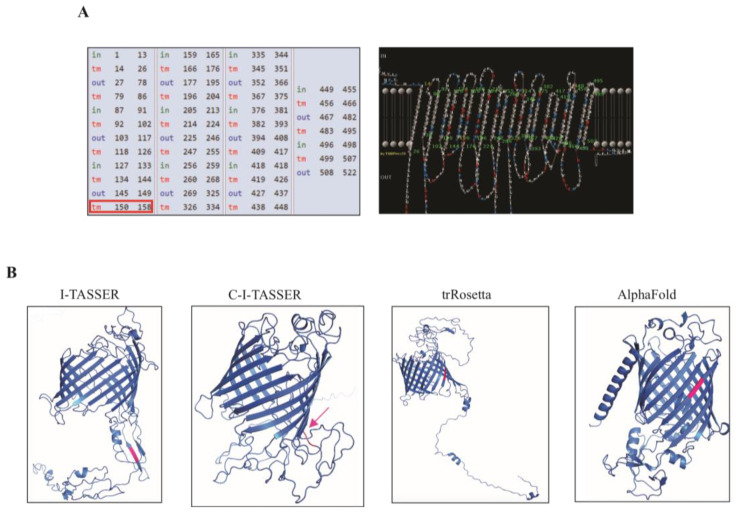
Structural characterization of LIC12254 by bioinformatics analysis. The topology of the β-barrel in the LIC12254 protein was predicted by PRED-TMBB, showing the location of each amino acid, i.e., extracellular (out), periplasmic (in) or transmembrane (tm), and a graphical representation demonstrating the relative position of the predicted transmembrane strands with respect to the lipid bilayer. The RGD motif position (153–155) is highlighted in red (**A**). The 3D model was generated by four different web servers and visualized by PyMOL. The RGD motif is highlighted in pink (**B**).

By using bioinformatics tools, the LIC12254 protein sequence was analyzed for prediction of SPI cleavage site, conserved domain, cellular localization and size in amino acids. According to the LipoP program, the predicted cleavage site for the LIC12254 protein is between amino acids 34 and 35, and the CELLO software predicted its location in the outer membrane of the bacteria. The Interprot web server showed the presence of two conserved domains in the protein sequence, a domain of unknown function (DUF5982) and an Omp85/surface bacterial antigen domain (Table 3). The β-barrel topology in the LIC12254 protein was predicted by PRED-TMBB. This web server showed the location of each amino acid, i.e., extracellular (out), periplasmic (in) or transmembrane (tm), as observed in Figure 1A (left panel). In addition, a graphical representation showing the relative position of the predicted transmembrane strands with respect to the lipid bilayer was achieved, and the presence of 21 beta strands located in the transmembrane region was predicted for LIC12254 (Figure 1A–right panel) [15].

The tree-dimensional structure prediction of LIC12254 was carried out using three different web servers, namely, I-TASSER, C-I-TASSER and TrRosetta. Apart from that, the LIC12254 (encoded by Q72Q59 according to UniProt) has a structure predicted by Alphafold2. The figures were generated using the PyMOL program (Figure 1B). All four web servers predicted a β-barrel structure and an irregular coil region, but the localization of the RGD motif was different. The I-TASSER web server showed the motif in a beta strand located outside the β-barrel, C-I-TASSER predicted the RGD motif in an irregular coil, and TrRosetta and Alphafold2 showed this sequence located in a beta strand of the β-barrel structure (Figure 1B). Despite the difference observed in the RGD motif predicted localization, the β-barrel structure present in the LIC12254 3D structure suggested that this protein is an outer membrane protein, corroborating its cellular localization prediction.

### 3.2. RGD Motif Conservation among Leptospiral Pathogenic and Non-Pathogenic Species

**Figure 2:**
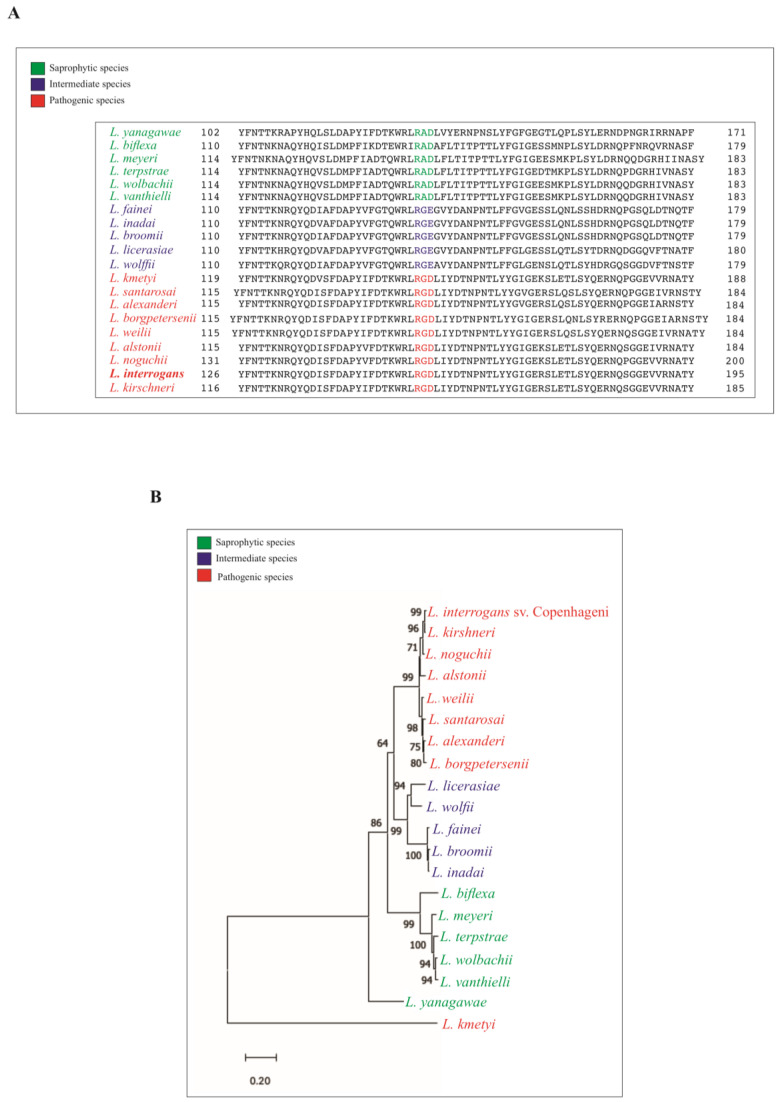
Sequence conservation among leptospiral pathogenic and non-pathogenic species. Sequence alignment was performed using the PROMALS3D tool. The RGD, RGE and RAD motifs are highlighted in pathogenic, intermediary and saprophyte strains, respectively (**A**). The phylogenetic tree was generated using the Neighbor-Joining method in the Mega11 software (**B**). The resulting phylogram shows a high degree of identity between the pathogenic species. The numbers at the nodes are bootstrap proportions in percent of 1000 replicates; scores lower than 60% were not considered.

To determine how conserved the RGD motif is among leptospiral groups, we carried out an extensive study using BLAST analysis. The LIC12254 protein sequences were submitted to GenBank, compared with the sequences in the database of nine pathogenic species (red), five intermediate species (blue) and six saprophytic species (green) of *Leptospira* [30], and the protein alignment was constructed with the sequences using the PROMALS3D alignment program. As observed in Figure 2A, all orthologs of the LIC12254 protein encoded by pathogenic species contain the RGD motif in their protein sequence. On the other hand, in intermediate and saprophyte species, the motif is altered to RGE and RAD, respectively (Figure 2A). The evolutionary history of the LIC12254 protein was inferred by using the Neighbor-Joining method. A phylogenetic tree was generated by Mega 11 software (Figure 2B). The percentage of replicate trees in which the associated taxa clustered together in the bootstrap test (1000 replicates) are shown next to the branches; only percentages higher than 60 were considered. This analysis was performed with amino acid sequences from 20 species of *Leptospira*, including pathogenic, intermediate and saprophyte species. The pathogenic group (shown in red), except for *L. kmetyi*, has the same evolutionary ancestor with high bootstrap percentage. The intermediate species (shown in blue) shares a common ancestor and history with the pathogenic strains. The saprophyte group (shown in green) also shares a common ancestor, except for *L. yanagawae*.

### 3.3. Expression of the Lic12254 Gene by RT-qPCR

**Figure 3:**
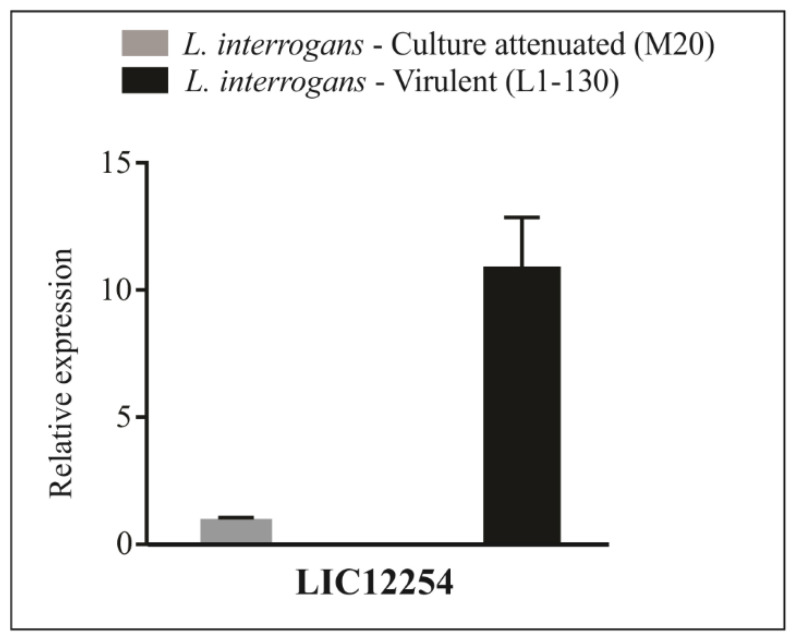
Transcript expression of the *lic12254* gene analyzed by RT-qPCR. The *lic12254* gene expression was analyzed in culture-attenuated (*L. interrogans* serovar Copenhageni, strain M20) and virulent (*L. interrogans* serovar Copenhageni, strain Fiocruz L1-130) strains by RT-qPCR. The result is the mean of three replicates and is representative of two independent experiments.

Gene expression in the virulent *L. interrogans* strain Fiocruz L1-130 and culture-attenuated *L. interrogans* strain M20 was compared by RT-qPCR. The results revealed the presence of LIC12254 mRNAs in both strains, with approximately a 10-fold increase in gene expression in the virulent *L. interrogans* L1-130 strain compared to the culture-attenuated *L. interrogans* M20 strain, suggesting the importance of LIC12254 in leptospiral virulence processes (Figure 3).

### 3.4. Cloning and Construction of the lic12254 Gene and the RGD Motif-Mutated lic12254 Sequence

**Figure 4:**
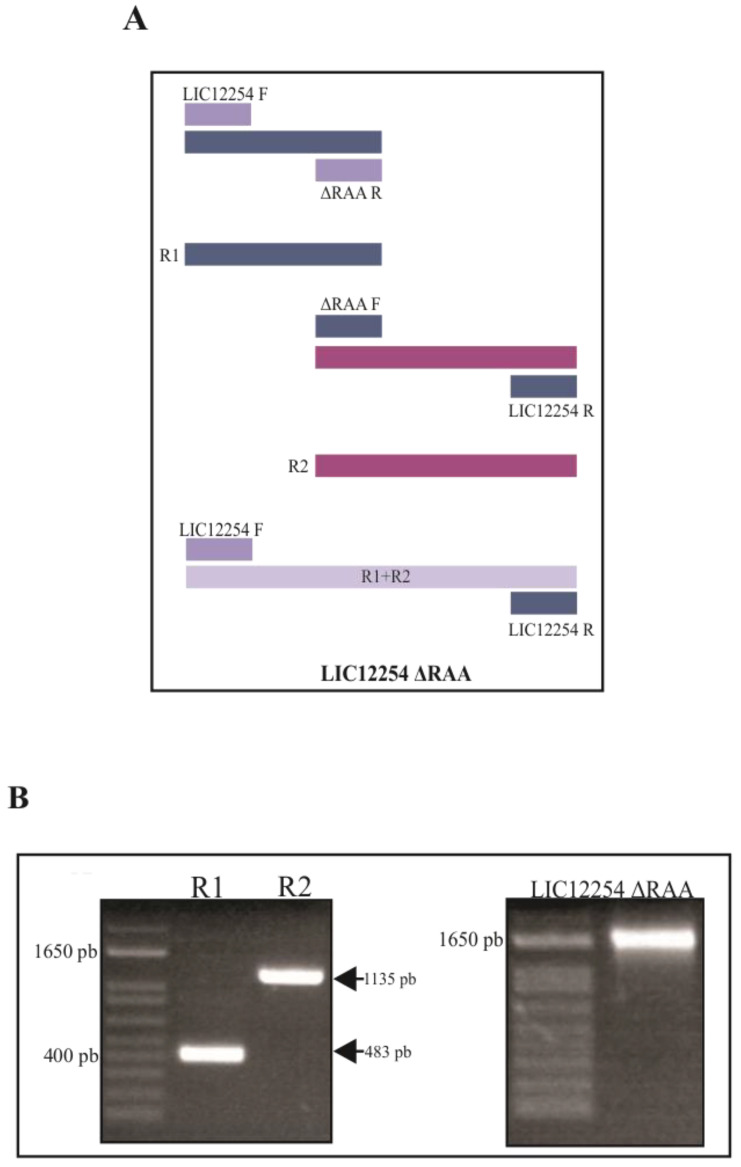
Construction of the RGD motif-mutated *lic12254* gene. Scheme of the RGD motif-mutated *lic12254* cloning. LIC12254 ΔRAA was generated from an oligonucleotide containing the sequence substitution corresponding to the replacement of RGD with RAA. After obtaining two PCR products, they were used as template for the amplification of the mutated *LIC12254* (**A**). Analysis of the PCR amplicons obtained in the constructions steps by 1% agarose gel electrophoresis (**B**).

The *lic12254* gene without the signal peptide was cloned into the expression vector pAE as described above. The RGD motif-mutated *lic12254* gene was obtained by PCR as shown in Figure 4A. Briefly, two PCR products were generated with a sequence substitution allowing the replacement of RGD with RAA. The products R1 and R2 were then analyzed by electrophoresis and found to be the correct size, i.e., 483 and 1135 bp, respectively. The PCR product resultant from R1 and R2 amplification was used as a template for another PCR round, and the final sequence of the RGD motif-mutated *lic12254* gene was obtained, with a length of 1570 bp (Figure 4B). This fragment was purified and cloned into the pAE expression vector, and the RGD motif mutation was confirmed by DNA sequencing (data not shown).

### 3.5. Production of the Recombinant Proteins in E. coli

**Figure 5:**
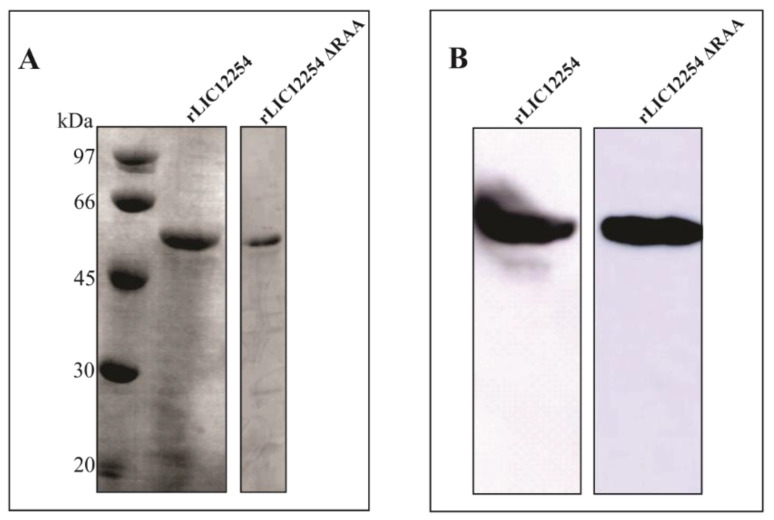
Analysis of the recombinant proteins by SDS-PAGE and Western blotting. Analysis of the rLIC12254 and rLIC12254 ΔRAA proteins in SDS-PAGE stained with Coomassie blue (**A**) recovered after urea solubilization. Western blotting analysis of rLIC12254 and rLIC12254 ΔRAA (**B**) using the anti-rLIC12254 antiserum produced in mice (1:2000) plus an horseradish peroxidase (HRP)- conjugated anti-mouse antibody (1:5000). Detection was performed using Super Signal West Dura Extended Duration Substrate (Thermo Fisher Scientific, Waltham, MA, USA).

Induction of unmutated and RGD motif-mutated *lic12254* gene expression was performed in *E. coli* BL21 Star (DE3) pLySs strain after addition of 1 mM IPTG. Both proteins were expressed in their insoluble form and purified. After in vitro refolding, the secondary structures of the recombinant proteins were assessed by circular dichroism analysis, and the unmutated and mutated proteins showed a majority profile of beta sheets structures (data not shown). As observed in Figure 5A, the rLIC12254 and rLIC12254 ΔRAA proteins were successfully achieved and showed an estimated molecular mass of 54 kDa. The recombinant LIC12254 and rLIC12254 ΔRAA (Figure 5B) proteins were confirmed by Western blot after probing with polyclonal antibodies generated in mice against rLIC12254.

### 3.6. Interaction of rLIC12254 with Human Integrins

**Figure 6:**
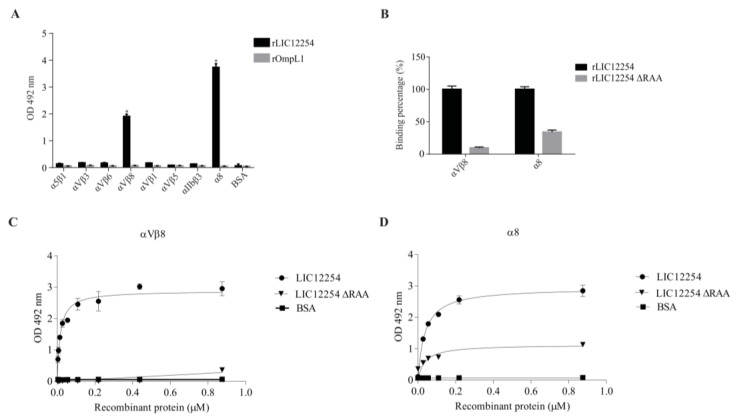
Binding of the recombinant proteins to the human integrins. ELISA plates were coated with human integrins or the control protein BSA. The recombinant proteins rLIC12254 and rOmpL1 (negative control) were added, and component binding was detected by incubation with anti-recombinant polyclonal antibodies (**A**). Bars represent the mean ± SD absorbance at 492 nm of three replicates and are representative of two independent experiments. For the statistical analyses, the interaction of the recombinant proteins with human integrins was compared to integrin binding to rOmpL1 (negative control) by two-tailed *t*-test (* *p* < 0.05). ELISA plates were coated with αVβ8 and α8 integrins or the control protein BSA. The recombinant proteins rLIC12254 and rLIC12254 ΔRAA were added, and binding was detected by incubation with anti-rLIC122254 polyclonal antibodies (**B**). Bars represent the mean ± SD absorbance at 492 nm of three replicates and are representative of two independent experiments. For comparative purposes, the absorbance of unmutated rLIC12254 was considered as 100% interaction. Dose-dependent binding experiments with the recombinant proteins and αVβ8 (**C**) and α8 (**D**) were performed by incubation with increasing recombinant protein concentrations. BSA was used as a negative control for non-specific binding. Binding was detected with an antiserum against rLIC12254. Each point represents the mean absorbance at 492 nm ± SD of three replicates and is representative of two independent experiments.

The integrin subtypes αvβ3, αvβ5, αvβ6, αvβ1, αvβ8, α5β1, αIIbβ3 and α8β1 were confirmed as receptors for RGD motif-containing adhesins from bacteria and viruses [31]. Thus, these eight integrins were tested as putative receptors for the leptospiral LIC12254 protein. As observed in Figure 6A, only αVβ8 integrin and the α8 chain bound to the rLIC12254. No binding was observed for the OmpL1 protein, used as negative control. When the binding of rLIC12254 ΔRAA to both integrins was tested, we observed a reduction in the binding of 90% for αVβ8 and of more than 65% for α8 (Figure 6B). The rLIC12254 protein was found to bind to the αVβ8 and α8 integrins in a dose-dependent manner (Figure 6C,D, respectively). Saturation was reached for both interactions with K*_D_* values of 31.55 ± 6.736 and 13.50 ± 2.424 nM for αVβ8 and α8, respectively. Likewise, rLIC12254 ΔRAA interacted with α8, though to a lower extent when compared to rLIC12254 (Figure 6C,D). These results indicated that the interaction of rLIC12254 with the αVβ8 and α8 integrins is mainly RGD-dependent.

## 4. Discussion

The successful establishment of infection by pathogenic microorganisms is attributed to their ability to adhere to, colonize and invade host tissues and to survive the host immune attack [32]. Pathogenic *Leptospira* spp. express a broad repertoire of proteins with the potential to participate in host adhesion [3]. Adherence occurs when bacterial adhesins recognize and bind to host cell receptors such as integrins and extracellular matrix (ECM) [33]. Integrins are a large protein family that plays a role important in several biological processes through binding to diverse ligands [31]. Among these ligands, many ECM components interact with integrins via the RGD motif, contributing significantly to cell communication. Aiming at interfering in the host cell machinery, many pathogens have protein sequences containing an RGD motif that can interact with integrins and mimic the role of these ligands. Thus, once activated, integrins can trigger intracellular signaling that causes cytoskeletal rearrangements and leads to microbial internalization [34]. Several pathogenic bacteria have been reported to utilize RGD motifs as virulence factors, including *Streptococcus*, *Bordetella pertussis*, *Helicobacter pylori* and *Mycobacterium* spp. [35,36,37,38,39,40,41].

In the case of spirochetes, it was shown that both the P66 and the BB0172 proteins present in *Borrelia burgdorferi* are able to interact with human integrins [42,43]. *Leptospira* spp. also have a repertoire of proteins containing RGD motifs. In 2012, Zhang and collaborators characterized the leptospiral protein Mce as an RGD motif-dependent adhesion and invasion factor. In the present study, we identified and characterized the function of an RGD motif present in the LIC12254 leptospiral protein sequence. According to the in silico analyses, this sequence is highly conserved among pathogenic strains, and the RGD motif is present only in pathogenic *Leptospira* strains, which suggests its involvement in bacterial virulence mechanisms. The 3D structure predictions showed that LIC12254 is a β-barrel protein. In Gram-negative bacteria, β-barrel transmembrane proteins, outer membrane proteins and lipid-anchored proteins are types of proteins that are surface-exposed [44]. A previous study characterized the leptospiral LIC12254 protein as a BamA-like βb-OMP protein, but the presence of an RGD motif in LIC12254 was not highlighted, and another domain identified as bacterial surface antigen D15/Oma87 was reported [45], suggesting that LIC12254 might have an even greater role in leptospiral pathogenesis. According to PRED-TMBB, the amino acids that compose the RGD motif are predicted to be localized in the transmembrane strand as part of the β-barrel. These data corroborated the 3D structure predicted by the TrRosetta web server but not the I-TASSER and C-I-TASSER prediction. The reason for such differences is unknown, but these localization differences have been reported for other pathogens such as *Helicobacter pylori* [46]. In these bacteria, the protein CagL has an RGD motif typically localized in exposed loop conformations but can also exist in helical regions.

Unlike the data reported for the leptospiral protein Mce that showed a high binding affinity to the integrins α5β and αVβ3 [13] and the integrins classified in family 2, αLβ2 and αMβ2 [47], our experiments showed that the rLIC12254 protein interacted significantly with the human integrins αVβ8 and α8. The major biological function of the αVβ8 integrin is the regulation of cell communication in tissues, but it is also involved in the control of intracellular signaling pathways [48]. It has been described that after the interaction of a protein from herpes simplex virus (HSV) with αVβ8 integrin, conformational changes occurred, leading to the endocytosis of HSV [49]. It is also speculated that αVβ8 is involved in severe acute respiratory syndrome coronavirus 2 (SARS-CoV-2). An RGD motif has been found in the spike protein sequence of SARS-CoV-2 but not in other coronaviruses. As an RGD motif is close to the angiotensin-converting enzyme 2 (ACE2) receptor, it is possible that an interaction between the spike protein and αVβ8 creates a cell surface complex with ACE2, allowing the entry of SARS-CoV-2 into cells [50]. Yet, it is not known if other receptors are involved in the interaction of rLIC12254 with integrins, but by mutating the recombinant protein RGD motif, we observed that the interaction was almost totally inhibited, suggesting that the leptospiral LIC12254 protein is an RGD motif-dependent integrin-binding protein.

Interestingly, the α8 integrin chain seems to play a central role in glomerular function, maintaining its integrity during glomerular injury. It is expressed on mesangial cells and regulates the properties of adhesion, proliferation and migration by interacting with fibronectin, vitronectin or osteopontin [51]. Recently, the α8 integrin was shown to be involved in the phagocytosis process mediated by renal mesangial cells, possibly through cytoskeleton interactions [52]. Since α8 is found in abundance in renal cells and since the kidneys are target sites for leptospiral colonization, it is possible that combination affords an interaction allowing leptospires to subvert host physiological processes in their favor.

In conclusion, our results showed that LIC12254 is a leptospiral putative outer membrane protein capable of interacting with human integrins via the RGD motif. Thus, LIC12254 could be involved in bacterial pathogenesis through the adhesion process. To the best of our knowledge, LIC12254 is the first leptospiral protein found to interact with human αVβ8 integrin and the and α8 integrin chain.

## Figures and Tables

**Table 1:** Species name and protein database codes.

Species Name	TaxID	NCBI Accession
*L. interrogans * sv. Copenhageni	267671	AAS70826
*L. kirschneri*	29507	WP_004766834.1
*L. noguchii*	28182	WP_002178526.1
*L. alstonii*	28452	WP_036040698.1
*L. weilii*	28184	WP_061223108.1
*L. santarosai*	28183	WP_004465014.1
*L. alexanderi*	100053	WP_078123768.1
*L. kmetyi*	408139	WP_020986420.1
*L. borgpetersenii*	174	WP_002740814.1
*L. licerasiae*	447106	WP_135668817.1
*L. fainei*	48782	WP_016549264.1
*L. broomii*	301541	WP_010570345.1
*L. inadai*	29506	WP_010411470.1
*L. wolffii*	409998	WP_135698917.1
*L. biflexa*	172	WP_135736477.1
*L. meyeri*	29508	WP_020776522.1
*L. terpstrae*	293075	WP_002972933.1
*L. wolbachii*	29511	WP_015682622.1
*L. vanthielii*	293085	WP_135659155.1
*L. yanagawae*	293069	WP_015677120.1

**Table 2:** Oligonucleotides used for cloning the *lic12254* and *lic12254Δ RAA* genes in the pAE vector.

Primer	Sequence (5′→3′)	Context of Use
LIC12254 F	ATCGCTCGAGCAAGAAGATTGTTCTAAG	Cloning
LIC12254 R	ATCGGGTACCATCGTCAGAAAATGTGATTAAAGTTC	
ΔRAA R	GTATTTGGATTCGTATCATAGATTAACGCAGCTCTTAGTCTCCATTTGGT	RGD replacement
ΔRAA F	CTTTGATACCAAATGGAGACTAAGAGCTGCGTTAATCTATGATACGAATC	
LIC12254 F	CCGTTTCCGAAGGTATTTGA	qPCR
LIC12254 R	GCAAAATGTTGTCCGGCTAT	
16S F	CACGAAAGCGTGGGTAGTGA	
16S R	CAACGTTTAGGGCGTGGATTA	
T7 F	TAATACGACTCACTATAGGG	Sequencing
T7 R	TAGTTATTGCTCAGCGGTGG	

**Table 3:** In silico analysis of the coding sequence of LIC12254.

Annotation (NCBI)	Cleavage Site (LipoP)	Domain Prediction (Interprot)	Cellular Localization (CELLO)	Size (aa)
Outer membrane protein	SPI 34–35	DUF5982 (56–119) Omp85/Surface bacterial antigen (128–511)	Outer membrane	522

## Data Availability

Not applicable.
